# Selecting Informative Patients for Phase 2 Progressive Trials in MS: Design Considerations for Phase 2 Clinical Trials in Progressive MS

**DOI:** 10.1177/13524585241274620

**Published:** 2024-09-08

**Authors:** Marcus W. Koch, Carlos Camara-Lemarroy, Eva Strijbis, Jop Mostert, Victoria M. Leavitt, Pavle Repovic, James D. Bowen, Jacynthe Comtois, Bernard Uitdehaag, Gary Cutter

**Affiliations:** Department of Clinical Neurosciences, University of Calgary, Calgary, AB, Canada; Department of Clinical Neurosciences, University of Calgary, Calgary, AB, Canada; Department of Neurology, MS Center Amsterdam, Amsterdam University Medical Center, Amsterdam, The Netherlands; Department of Neurology, Rijnstate Hospital, Arnhem, The Netherlands; Department of Neurology, Columbia University Irving Medical Center, New York, NY, USA; Multiple Sclerosis Center, Swedish Neuroscience Institute, Seattle, WA, USA; Multiple Sclerosis Center, Swedish Neuroscience Institute, Seattle, WA, USA; Department of Medicine, Neurology Service, Hôpital de la Cité-de-la-Santé, Laval, QC, Canada; Department of Neurology, MS Center Amsterdam, Amsterdam University Medical Center, Amsterdam, The Netherlands; Department of Biostatistics, The University of Alabama at Birmingham, Birmingham, AL, USA

**Keywords:** Clinical trial, outcome measurement, progressive, disease modifying therapies, atrophy

## Abstract

While relapsing-remitting multiple sclerosis (MS) has many therapeutic options, progressive forms of MS remain largely untreatable. Phase 2 clinical trials are our main tool to advance new treatments for progressive MS. Given the complexities of progressive MS, it will likely require many phase 2 trials to improve its treatment. To conduct informative and efficient phase 2 trials, it is important that such trials are designed in a way that they can identify a successful treatment as quickly and with as few participants as possible. In this topical review, we discuss cohort selection, outcome selection, cohort enrichment, and dosing selection as strategies to optimize the efficiency of phase 2 clinical trials in progressive MS.

## Introduction

While the treatment of relapsing-remitting multiple sclerosis (RRMS) has been transformed by the advent of immunomodulatory treatments, progressive MS remains largely untreatable. People with secondary or primary progressive MS (SPMS, PPMS) experience a steady and unrelenting worsening of disability, which current treatments do not meaningfully affect. Many more clinical trials in progressive MS will likely be necessary to discover successful treatments. In this topical review that is based on a presentation at the ACTRIMS Forum 2024 conference, we review strategies to optimize the efficiency of phase 2 clinical trials in progressive MS.

**Figure 1.**
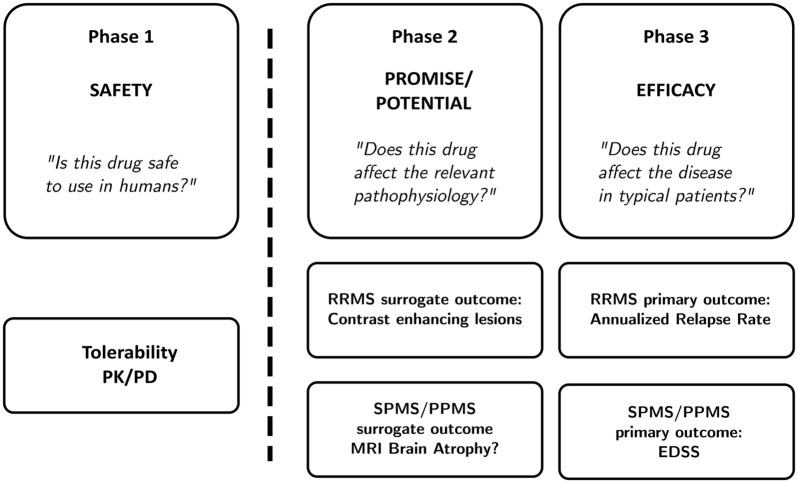
A review of clinical trial phases. Phase 1 trials are concerned with the safety of treatments, and therefore do not investigate a treatment effect in the disease of interest. Phase 2 trials investigate whether a drug can affect the relevant pathophysiology, and phase 3 trials determine whether a drug works in typical people with the disease. Phase 2 trials often use surrogate outcomes or biomarkers of response as their primary outcome: contrast enhancing lesions in RRMS, and MRI brain atrophy in progressive MS.

Figure 1 gives an overview of classical trial phases. Phase 1 trials generally build on animal studies and are meant to establish the general safety and tolerability of treatment in humans. They are often small first-in-human studies in healthy volunteers and can include measurements of pharmacokinetics and pharmacodynamics. Phase 1 trials answer the question: “Is this drug safe to use in humans,” and the primary outcome is, therefore, the safety and tolerability of the treatment. Once the immediate safety of a treatment is established, the drug is tested for its effect on relevant disease processes in a phase 2 trial.

Phase 2 trials are crucial steps in discovering new treatments. Unfortunately, treatments for progressive MS have historically not been developed using these classical trial phases. Rather, phase 3 trials in progressive MS (such as the INFORMS trial of fingolimod in PPMS including 970 participants,^1^ the ASCEND trial of natalizumab in SPMS including 889 participants,^2^ and the ORATORIO trial of ocrelizumab in PPMS including 732 participants^3^) used drugs with demonstrated treatment effects in RRMS in people with a progressive disease course without conducting a separate phase 2 trial. This practice, which may be based on the assumption that RRMS and progressive MS are driven by similar disease processes, has proven suboptimal in the search for effective treatments, and may in fact have contributed to our failure.

In the interest of performing efficient and informative phase 2 trials (since many trials at this stage will be negative), researchers try to limit the cost of phase 2 trials by limiting their size and duration. One strategy to allow for shorter and smaller trials is the use of a surrogate outcome. In RRMS, for example, we are ultimately interested in finding a medication that prevents MS relapses. Since relapses occur relatively infrequently, it has become customary to use the incidence of new contrast enhancing lesions (CELs) rather than the number of relapses as the primary outcome measure in phase 2 trials in RRMS. This strategy is based on the idea that CELs and relapses are part of the same disease process and so closely related that CELs can “stand in” for clinical relapses (in the sense that it is reasonable to expect that a treatment that prevents CELs also prevents clinical relapses).^4^

Thus, a phase 2 trial asks: “Does this drug affect the relevant pathophysiology?” Phase 3 trials ask: “Does this drug affect the disease course in typical patients?” Because phase 3 trials use clinically meaningful outcomes, results are more meaningful if inclusion criteria match a real-world patient population. In the framework of phase 2 trials in progressive MS, “informative patients” can refer to several aspects of trial design. In this review, we will focus on four of them: (1) cohort selection, (2) outcome selection, (3) cohort enrichment, and (4) dosing selection.

## Informative Patients I: Choosing the Right Trial Cohort

Since phase 2 trials are meant to inform us about the effect of a treatment on a relevant disease mechanism, it is legitimate to use specific inclusion and exclusion criteria to select for an informative trial cohort. For example, in a phase 2 futility trial in PPMS, we were interested in selecting a group of participants with minimal focal inflammatory disease activity, and decided to exclude participants with CELs at screening.^5^ Similarly, it has recently become better documented that focal inflammatory disease activity in RRMS decreases with advancing age.^6–8^ Researchers interested in finding a new treatment for RRMS could reason that the focal inflammatory disease activity they are interested in reducing is more commonly present in younger patients, and decide to include only young participants in their phase 2 trial. We find this focus on a specific “informative” trial cohort legitimate, provided that the researchers subsequently include “typical” participants, in this case people across the entire age spectrum seen in clinical practice, in their confirmatory phase 3 trial.

One difficulty of translating phase 3 clinical trial findings into treatment recommendations is the question whether the included trial participants are a good representation of the patient population. For example, CELs were present at baseline in 27.5% (treatment arm) and 24.7% (placebo arm) of participants in ORATORIO, a positive phase 3 trial of ocrelizumab treatment in PPMS,^3^ which is much higher than in previous phase 3 trials in PPMS (INFORMS: 13%,^1^ PROMISE: 14.1%^9^). This is relevant because ocrelizumab is a highly successful treatment in RRMS that prevents relapses and magnetic resonance imaging (MRI) activity and therefore likely has a greater treatment effect in PPMS patients with remaining focal inflammatory disease activity. Therefore, since ORATORIO included twice as many PPMS patients with CELs at baseline as previous comparable trials, it is unclear whether the small but statistically significant treatment effect of ocrelizumab in this trial translates into a benefit for typical PPMS patients. To make sure the trial cohort represents typical patients seen in clinical practice, investigators should consider including participants up to the age of 65, and to limit the inclusion of people with CEL at baseline to population levels.

## Informative Patients II: Choosing Outcome Measures

Trial participants can only be “informative” by the change they exhibit on an outcome measure. In other words, we can only decide that a treatment for progressive MS works if it improves the outcome or demonstrates slower worsening on the outcome. Therefore, “informativeness” largely depends on the selection of appropriate outcome measures that are sensitive to change. Figure 1 shows the current approach to measuring outcomes in RRMS and progressive MS. In RRMS, we often use CELs as an outcome measure in phase 2 trials with the underlying reasoning that a treatment that reduces CELs likely also reduces clinical relapses (see Figure 1). Phase 2 trials in progressive MS to date have either used worsening on the Expanded Disability Status Scale (EDSS)^10^ or MRI brain atrophy as their primary outcomes. In progressive MS, we are ultimately interested in a treatment that prevents the steady worsening of disability that people with progressive MS experience. Because it is thought that measuring disability directly either takes too long, or the measurement is too unreliable, the outcome of MRI brain atrophy, either of the whole brain or regions thereof, is now commonly used as the primary outcome in phase 2 trials in progressive MS.^11,12^ The underlying reasoning here is that the disease process of progressive MS leads to worsening disability as well as to brain atrophy, and that a measurement of brain atrophy can “stand in” for a measured worsening of disability, similar to how CELs can “stand in” for clinical relapses in RRMS. One problem with this approach is that while both brain atrophy and disability increase with time, they have not been shown to correlate well and may both be mere functions of biological aging. In addition, there are debates over the best way to measure atrophy, especially regional brain atrophy. Finally, the interpretation of brain atrophy measures is complicated by technical issues, such as pseudoatrophy, diurnal fluctuations, and hydration state.^13^

**Figure 2.**
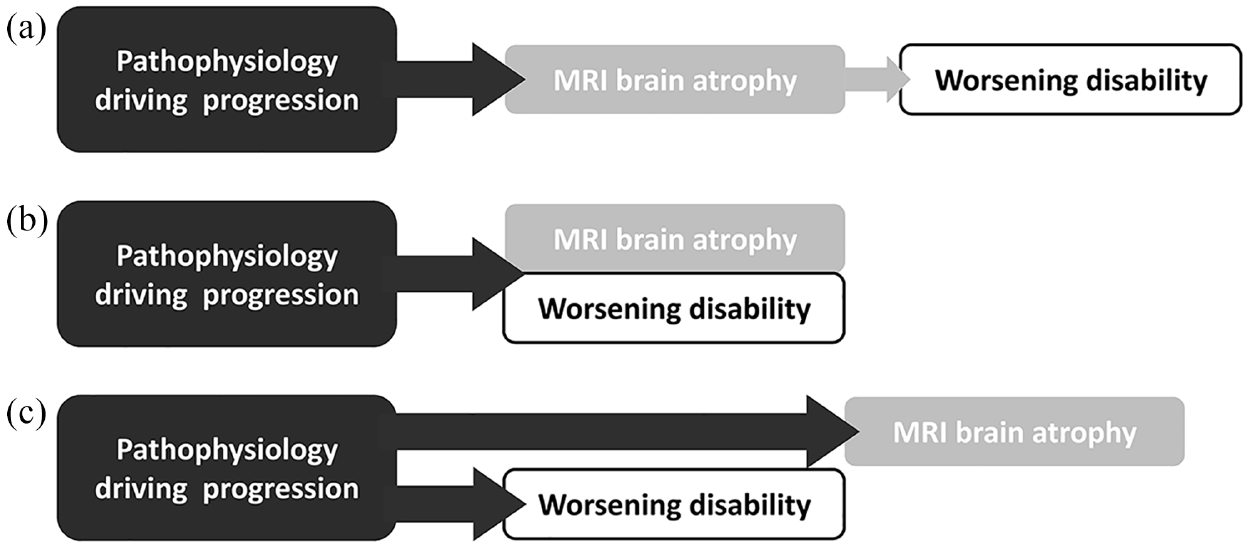
Problems with surrogate outcomes in progressive MS phase 2 trials. Here, we use MRI brain atrophy as an example, but the overall concept is true for all surrogate outcomes. (a) Shows the ideal situation: MRI brain atrophy as the surrogate outcome precedes and predicts disability worsening. In this case, MRI brain atrophy can indeed “stand in” for physical disability. (b) Shows an intermediate scenario: here the disease process of progression drives both MRI brain atrophy and physical disability. In this case, it is possible to use MRI brain atrophy as the outcome, but it is not necessary, as it would be easier to measure physical disability directly. (c) shows the worst-case scenario: both MRI brain atrophy and physical disability are driven by the same pathophysiology, but there is no close relationship between the two, and one does not predict the other.

Figure 2 shows an overview of the current approach: if MRI brain atrophy precedes and perfectly predicts disability worsening (see Figure 2(a)), MRI brain atrophy would indeed be a useful surrogate outcome, especially if it can be identified more quickly, easily, or reliably than physical disability. However, it is also possible that MRI brain atrophy and disability worsening occur over the same time course and in a similar degree (Figure 2(b) shows this intermediate scenario), in which case there is no advantage to measuring MRI brain atrophy over measuring disability directly. In this scenario, it would be more straightforward (and cheaper) to measure physical disability directly. Figure 2(c) shows the worst-case scenario: the disease process of progressive MS causes both physical disability and MRI brain atrophy, but there is no clear relation between the two in timing and degree. In our investigation in the ASCEND trial (a large phase 3 trial in SPMS),^2^ we found no significant associations between disability worsening and several MRI brain atrophy measures over 2 years of follow-up,^14^ which calls into question the use of this outcome as a surrogate. It is also worth mentioning in this context, that in the phase 2 trials of simvastatin in SPMS and of ibudilast in SPMS/PPMS, MRI brain atrophy outcomes and serum biomarkers of neuroaxonal injury were discordant.^15,16^ We are focusing on MRI brain atrophy here because it is the most widely used outcome, but the same thinking applies to other outcome measures such as retinal atrophy, or serum or CSF biomarkers: the measurement of serum biomarkers such as Neurofilament light chain, for example, is not standardized, which makes the interpretation of this biomarker difficult, especially when comparing between studies;^17^ similarly, advanced retinal atrophy measures such as ganglion cell + inner plexiform layer (GCIPL) thickness depend on imaging processing techniques that are not yet standardized.^18^ The MRI has the benefit that it is extremely precise and can detect very small differences in relatively small sample sizes, but it may be detecting differences that are not clinically meaningful. Thus, while it is a precise outcome measure, it may not be an informative one.

Ultimately, a successful treatment for progressive MS should reduce the accumulation of disability, or ideally reverse disability. The measurement of physical disability should, therefore, be the most important aspect to consider when selecting informative trial outcomes. The EDSS^10^ is the most commonly used measure of physical disability, despite its considerable flaws. In SPMS, Koch et al.^19^ and Ebers et al.^20^ have shown that the number of worsening events on the EDSS over time is similar to the number of improvements by the same margin, which suggests that measurement error is driving the changes and that the EDSS is not a reliable measure of disability in SPMS. Interestingly, the timed 25-foot walk (T25FW) avoids these problems:^19^ on the T25FW, worsening events outnumber improvement events in progressive MS, and the T25FW records more worsening events over time than the EDSS. We, therefore, recommended it as a more appropriate outcome for phase 2 and phase 3 trials in progressive MS. Others have shown that T25FW changes precede changes on the EDSS, so that T25FW changes can “stand in” for EDSS changes (the situation pictured in Figure 2(a)).^21^

## Informative Patients III: Enriching the Trial Sample

**Figure 3.**
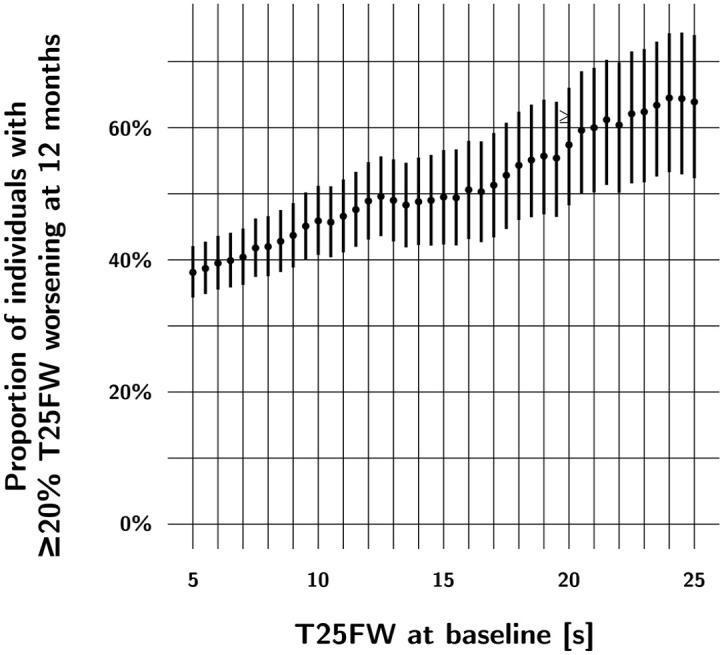
In SPMS, the percentage of people with significant (⩾ 20%) worsening in T25FW performance at 12 months increases as a function of baseline T25FW performance in this cohort of patients with baseline EDSS scores between 4.0 and 6.5. This cohort combines participants of two clinical trials and real-world data (*n* = 235; adapted from Koch et al.^22^). This circumstance allows for the “enrichment” of trial populations: for example, by including people with a baseline T25FW performance of 7 seconds or more, we would expect 40% of this cohort to experience significant worsening at 12 months. If we include participants with a baseline T25FW performance of 12 seconds or more, the percentage is increased to almost 50% of the cohort.

Another method to select for “informative” patients depends on the selected primary outcome measure. In SPMS, for example, we learned from a comparison of T25FW measurements in two trial datasets and real-world clinical data that the percentage of trial participants with significant disability worsening increases as a function of baseline performance on the T25FW and EDSS.^22^ We can use this characteristic of the T25FW to aim for an expected rate of worsening. Among participants with a baseline EDSS score of between 4.0 and 6.5, we found that the percentage of participants with significant worsening of disability at 12 months (defined as ⩾ 20% worsening on the T25FW) was 38.1% for participants with a baseline T25FW ⩾ 5 seconds, 43.7% in those with a baseline T25FW ⩾ 9 seconds, and 49% in those with a baseline T25FW ⩾ 15 seconds (see Figure 3). This strategy can be used to reduce the sample size for a phase 2 trial in progressive MS. We found no comparable strategy that could be used with the EDSS.^22^ However, while it is attractive to enrich a trial cohort in this way, such enrichment strategies should be applied carefully, as there is a trade-off between creating a more homogeneous trial population and the generalizability of the trial results. Another aspect to keep in mind is the possible increase in variance in “enriched” trial populations as these are often from the more extreme ranges of the variable. The variance is the average squared difference from the mean and if we select for patients on the extremes of baseline performance, this can also increase the variance of the measured outcomes. This can ultimately increase the risk of a type 2 error (the risk of falsely rejecting a useful treatment or “false negative”).

## Informative Patients IV: Dose-Finding in Phase 2 Trials

Finally, phase 2 trials can be informative in the sense that they can define the standard dose of the treatment under investigation. This often means including a high dose and a low dose to understand the benefits and risk of higher doses relative to side effects and serious adverse events. While the basic safe dose information of a new treatment comes from the phase 1 trial, and the ultimate question whether the treatment is safe and tolerable in typical patients should be explored in phase 3, phase 2 trials can help decide between choices for a standard dose. This is also important because dose-finding at the phase 3 trial is extremely costly and exposes a large number of participants to potential inefficacy or harm. A treatment should be dosed high enough that it has a meaningful treatment effect on the underlying disease process in typical patients. On the contrary, side effects of treatments normally increase with increasing doses, so that a treatment should also be dosed low enough that it is tolerable for typical patients. Phase 2 studies can address this dose-finding question in a limited way, by including additional treatment arms to the trial design. In RRMS, for example, the phase 2 FREEDOMS trial of fingolimod compared CELs between placebo, 0.5 mg fingolimod, and 1.25 mg fingolimod arms.^23^ Using this design, the trialists were able to see that both fingolimod doses had similar efficacy, but that the higher fingolimod dose was associated with more adverse events, which contributed to the selection of 0.5 mg as the standard dose. While most phase 2 trials in progressive MS compare one candidate treatment to placebo, the ARPEGGIO trial of laquinimod in PPMS similarly included three trial arms (placebo, laquinimod 0.6 mg, laquinimod 1.5 mg).^24^

## Conclusion

Phase 2 trials are our main tool to discover successful treatments in progressive MS. Fortunately, there is increasing interest in the design of phase 2 trials, either as single trials or combined in multi-arm platform trials,^25^ and more availability of funds for such studies. Based on our current knowledge, we recommend the following strategies to improve the design of phase 2 trials in progressive MS: (1) excluding participants with CELs at baseline or limiting their inclusion to population levels (informative trial cohort), (2) using the T25FW rather than the established EDSS as the primary clinical outcome measures (informative outcome measure), and (3) including participants with T25FW baseline performance above a certain cut-off (enrichment of the trial cohort).
